# Expression of the Stress Response Oncoprotein LEDGF/p75 in Human Cancer: A Study of 21 Tumor Types

**DOI:** 10.1371/journal.pone.0030132

**Published:** 2012-01-19

**Authors:** Anamika Basu, Heather Rojas, Hiya Banerjee, Irena B. Cabrera, Kayla Y. Perez, Marino De León, Carlos A. Casiano

**Affiliations:** 1 Center for Health Disparities and Molecular Medicine and Department of Basic Sciences, Loma Linda University School of Medicine, Loma Linda, California, United States of America; 2 Department of Pathology and Human Anatomy, Loma Linda University School of Medicine, Loma Linda, California, United States of America; 3 Division of Biostatistics and Epidemiology, Medical University of South Carolina, Charleston, South Carolina, United States of America; 4 Department of Medicine, Loma Linda University School of Medicine, Loma Linda, California, United States of America; Louisiana State University and A & M College, United States of America

## Abstract

Oxidative stress-modulated signaling pathways have been implicated in carcinogenesis and therapy resistance. The lens epithelium derived growth factor p75 (LEDGF/p75) is a transcription co-activator that promotes resistance to stress-induced cell death. This protein has been implicated in inflammatory and autoimmune conditions, HIV-AIDS, and cancer. Although LEDGF/p75 is emerging as a stress survival oncoprotein, there is scarce information on its expression in human tumors. The present study was performed to evaluate its expression in a comprehensive panel of human cancers. Transcript expression was examined in the Oncomine cancer gene microarray database and in a TissueScan Cancer Survey Panel quantitative polymerase chain reaction (Q-PCR) array. Protein expression was assessed by immunohistochemistry (IHC) in cancer tissue microarrays (TMAs) containing 1735 tissues representing single or replicate cores from 1220 individual cases (985 tumor and 235 normal tissues). A total of 21 major cancer types were analyzed. Analysis of LEDGF/p75 transcript expression in Oncomine datasets revealed significant upregulation (tumor vs. normal) in 15 out of 17 tumor types. The TissueScan Cancer Q-PCR array revealed significantly elevated LEDGF/p75 transcript expression in prostate, colon, thyroid, and breast cancers. IHC analysis of TMAs revealed significant increased levels of LEDGF/p75 protein in prostate, colon, thyroid, liver and uterine tumors, relative to corresponding normal tissues. Elevated transcript or protein expression of LEDGF/p75 was observed in several tumor types. These results further establish LEDGF/p75 as a cancer-related protein, and provide a rationale for ongoing studies aimed at understanding the clinical significance of its expression in specific human cancers.

## Introduction

A growing body of evidence supports the central hypothesis that an augmented state of cellular oxidative stress (ASCOS) is a major contributing factor to carcinogenesis [1], [2]. Possible triggers of ASCOS include lifestyle and environmental-related factors such as diet, infections, cigarette smoking, alcohol, and pollutants [3]. ASCOS causes damage to DNA, protein, and lipids, as well as activation of stress transcription factors, leading to the activation of stress, antioxidant, inflammatory, and pro-survival pathways that contribute to malignant transformation, cell cycle deregulation, resistance to cell death and therapy, invasion, angiogenesis, and metastasis [1], [2].

LEDGF/p75 is a stress response protein that promotes cell survival in the presence of environmental stressors that induce ASCOS, such as chemotherapy, radiation, heat, and serum starvation [4]–[7]. LEDGF/p75 is also known as transcription co-activator p75 [8], PC4 and SFRS1 interacting protein (PSIP1) [9], and dense fine speckled autoantigen of 70 kD (DFS70) [10]. It has been implicated in inflammation, autoimmunity, HIV-1 replication, and cancer [10]–[17]. The stress survival properties of LEDGF/p75 appear to be linked to its ability to bind specific transcription factors and facilitate the transactivation of stress, inflammatory, antioxidant, and cancer-associated genes [18]–[23]. LEDGF/p75 (530 amino acids) and its less characterized alternative splice variant, LEDGF/p52 (333 amino acids), are derived from the LEDGF/PSIP1 gene [8], [9], [24]. LEDGF/p52 corresponds to N-terminal amino acids 1–325 of LEDGF/p75, and contains an additional intron-derived 8 amino-acid tail [25]. Our group reported previously that LEDGF/p75 is upregulated in cancer cells compared to normal cells, and that LEDGF/p52 is expressed at relatively low levels in cancer cells, induces apoptosis when ectopically expressed, and antagonizes the pro-survival functions of LEDGF/p75 [7], [16], [25].

Several lines of evidence support the recent emergence of LEDGF/p75 as an oncoprotein in human cancer. For instance, LEDGF/p75 is a target of chromosomal translocations in leukemias, resulting in LEDGF/NUP98 fusion proteins with enhanced activity [23], [26]–[29]. Its mRNA expression was found to be upregulated in blasts from chemotherapy-resistant acute myelogenic leukemia patients, and its ectopic overexpression protected leukemia cells against drug-induced cell death [30]. LEDGF/p75 tethers the menin-mixed lineage leukemia (MLL) transcription factor complex to chromatin to activate leukemogenesis [23]. Our group reported previously that LEDGF/p75 is an autoantigen in prostate cancer (PCa), with elevated protein expression in prostate tumor tissues [16]. We also observed that its ectopic overexpression promoted cellular survival against stress-induced death in HepG2 liver tumor cells [7], and attenuated docetaxel-induced lysosomal cell death in PCa cells [31]. Daugaard et al. [17] reported elevated LEDGF/p75 transcript expression in human breast and bladder cancers, and that its ectopic overexpression in breast cancer cells protected against drug-induced lysosomal cell death and increased the tumorigenic potential of the cancer cells in murine models. Recently, elevated gonadotropins were shown to enhance LEDGF/p75-dependent activation of VEGF-C expression, augmenting lymphangiogenesis and angiogenesis of ovarian tumors [32].

Although the transcript expression of LEDGF/p75 was reported to be upregulated in leukemia, breast, and bladder cancers [17], [30], immunohistochemical (IHC) expression analysis of the protein in human tumor tissues has been performed only for PCa [16]. The emerging role of LEDGF/p75 as a stress survival oncoprotein led us to undertake a comprehensive analysis of its mRNA and protein expression in 21 major human tumor types, using cancer gene microarray databases, TissueScan Cancer Q-PCR array, and IHC analysis of tissue microarrays (TMAs). This study provides evidence that LEDGF/p75 is selectively upregulated in human cancers.

## Materials and Methods

### Bioinformatics Analysis of Oncomine Cancer Gene Microarray Database

For comparison of LEDGF mRNA expression between tumor and normal tissues, we selected datasets from the Oncomine database (Compendia Biosciences; Ann Arbor, MI, USA; www.oncomine.org). These datasets, containing gene microarrays of cancers and corresponding disease-free normal and/or normal adjacent tissues, provided fold-change data for gene expression (tumor vs normal), with p-values calculated by t-tests. It should be mentioned that the datasets examined were not specific for LEDGF/p75 but provided gene expression data for the LEDGF/PSIP1 gene, which gives rise to both the p75 and p52 splice variants. A total of 81 datasets were examined for LEDGF/PSIP1 transcript expression for the following cancers: bladder (2 datasets), breast (9 datasets), cervix (2 datasets), colon and rectum (3 datasets), esophagus (3 datasets), kidney (6 datasets), head and neck (8 datasets), liver (2 datasets), lung (9 datasets), lymphoma (3 datasets), ovary (6 datasets), pancreas (6 datasets), prostate (13 datasets), salivary gland (1 dataset), skin (5 datasets), stomach (2 datasets) and uterus (1 dataset). No microarray data was available for LEDGF/PSIP1 transcript expression in cancers of the gall bladder, small intestine, and thyroid.

### Human ‘TissueScan Cancer Survey Panel’ Q-PCR Array

We used the human ‘TissueScan Cancer Survey Panel 96–I’ Q-PCR array (CSRT-101, OriGene Technologies Inc., Rockville, MD, USA) for in-house analysis of LEDGF/p75 mRNA expression in 96 tissues covering 8 major human cancers (breast, colon, kidney, liver, lung, ovarian, prostate, and thyroid). This array consisted of first-strand complementary DNA (cDNA) from 9 individual cases of each cancer type and 3 cases of corresponding normal adjacent tissues. The manufacturer provided limited patient clinicopathological information (age, sex, tumor stage, pTNM stage), with no patient identifiers.

Q-PCR was performed in 96-well PCR array plates (OriGene) using the MyiQ thermal cycler (Bio-Rad Laboratories, Hercules, CA, USA) with primers specific for the LEDGF/p75 splice variant (forward 5′-TGCTTTTCCAGACATGGTTGT-3′ and reverse 5′-CCCACAAACAGTGAAAAGACAG-3′), and iQ SYBR Green Supermix (Bio-Rad). Briefly, 30 µl of PCR mixture, which included 15 µl of 2× master mix, 13 µl of double distilled water, and 1 µl each of forward and reverse primers (10 pmol/µl), were added to each of the 96 wells of the PCR array plates. PCR amplification was conducted at 95°C for 10 minutes, followed by 40 cycles of 95°C for 15 seconds and 60°C for 1 minute. mRNA expression was normalized using the expression of housekeeping gene human beta-actin, whose primers (forward 5′-CAGCCATGTACGTTGCTATCCAGG-3′ and reverse 5′-AGGTCCAGACGCAGGAT GGCATG-3′) were provided in the TissueScan Cancer Q-PCR array kit at a concentration of 10 pmol/µl. For data analysis, the ΔΔCt method was used. Fold changes were calculated as the difference in gene expression between tumors and corresponding normal adjacent controls.

### RNA Interference-mediated Knockdown of LEDGF/p75

PC-3 prostate cancer cells were purchased from American Type Culture Collection and cultured as recommended by the supplier in a humidified incubator with 5% CO_2_ at 37°C. Transient knockdown of LEDGF/p75 in PC-3 cells was carried out using the Amaxa Nucleofection method (Amaxa, Lonza) as described previously [21]. LEDGF/p75 siRNA (siLEDGF/p75) and the scrambled siRNA duplex (siSD, negative control) were synthesized by Integrated DNA Technologies (IDT). The siLEDGF/p75 sequence corresponded to nucleotides 1340–1360 (5′- AGACAGCAUGAGGAAGCGAdTdT-3′) with respect to the first nucleotide of the start codon of the LEDGF/p75 open reading frame [21]. This sequence corresponds to a region in the C-terminus of LEDGF/p75 not shared by LEDGF/p52. The sequence for siSD was 5′- GCGCGCUUUGUAGGAUUCGdTdT-3′ [21].

### Antibodies and Immunoblotting

The following antibodies were used: mouse monoclonals anti-β-actin (Sigma-Aldrich), and anti-LEDGF (1∶1000, BD Biosciences); rabbit polyclonals anti-LEDGF/p75 (1∶1000, Novus Biologicals); anti-LEDGF/p75 (1∶1000, Bethyl); and horseradish peroxidase (HRP)-labeled secondary IgG antibodies (Zymed). We also used a LEDGF/p75-specific rabbit polyclonal antibody that was developed at the W.M. Keck Autoimmune Disease Center of The Scripps Research Institute (La Jolla, CA, USA). This rabbit antibody, originally designated anti-DFS/LEDGFp75#5087 antibody (hereafter referred to as Scripps-Ab5087), was raised against a recombinant, truncated DFS70 protein generated from a partial cDNA clone, pDFS6.1, which encoded the C-terminal region of LEDGF/p75 [10]. To identify an antibody that is specific only for the LEDGF/p75 splice variant, we examined the reactivity of all the above-mentioned commercial and non-commercial LEDGF antibodies by immunoblotting using cell lysates from PC-3 cells with siSD and siLEDGF/p75.

Immunoblotting was carried out essentially as described previously [21]. Briefly, total proteins from PC3 cells with siSD and siLEDGF/p75 were separated by SDS-PAGE (NuPAGE 4–12%, Invitrogen) followed by transfer to polyvinyl difluoride (PVDF) membranes (Millipore). Membranes were blocked with 5% dry milk solution in TBS-T buffer (20 mM Tris-HCl, pH 7.6, 140 mM NaCl, 0.1% Tween 20) for 1 h and probed with primary antibodies. After several washes with TBS-T, membranes were incubated with horseradish peroxidase (HRP)-conjugated secondary antibodies for 30 minutes and then washed again with TBS-T. Protein bands were detected by enhanced chemiluminescence (Amersham).

### Immunohistochemical Analysis of Cancer Tissue Microarrays

Human cancer tissue microarrays (TMAs), commercially available from National Disease Research Interchange (NDRI, Philadelphia, PA, USA), Imgenex Corp. (San Diego, CA, USA) and US Biomax Inc. (Rockville, MD, USA), were used for IHC analysis of LEDGF/p75. Several different TMAs were used to increase the number of tumor tissue types and sample size (Table 1). Briefly, we acquired NDRI's comprehensive pan-cancer TMAs containing duplicate cores of 642 unique cases (522 tumor tissues of 20 major tumor types and 120 disease-free normal and/or normal adjacent specimens) in a 5 slide set (TS-1, TS-2, TS-3, TS-4 and TMA5). Each of pan-cancer TMAs IMH-326, IMH-327 and IMH-328 (Imgenex Corp), contained 59 specimens of various tumor types, while TMAs IMH-336, IMH-337 and IMH-338 (Imgenex Corp.) contained the corresponding 59 matched normal adjacent tissues.

**Table 1:** TMAs used for immunohistochemical analysis of LEDGF/p75 protein expression in tumors.

TMA	Source	Description	Tissue type	# of	# of	Total #
				cases	cores	of tissues
**TMAs with tumor tissues**						
TS-1	NDRI	Pan-cancer	Breast, ovarian, endometrial, gynecological	125	250	250
			tumors			
TS-2	NDRI	Pan-cancer	Lung, head and neck, colorectal, stomach,	139	278	278
			pancreatic tumors			
TS-3	NDRI	Pan-cancer	Bladder, kidney, prostate, testes tumors	129	258	258
TS-4	NDRI	Pan-cancer	Endocrine tumors including thyroid,	129	258	258
			lymphomas, sarcomas, melanomas			
IMH-	Imgenex	Pan-cancer	Stomach, esophagus, lung, colorectal,	59	59	59
326			thyroid, kidney tumors			
IMH-	Imgenex	Pan-cancer	Breast, liver, bladder, ovary, pancreas,	59	59	59
327			prostate tumors			
IMH-	Imgenex	Pan-cancer	Tumors of uterus, gall bladder, larynx,	59	59	59
328			uterine cervix, malignant lymphoma,			
**TMAs with normal tissues**						
TMA-5	NDRI	Pan-normal	Normal tissues corresponding to TS1-4	120	240	240
IMH-	Imgenex	N. Adj of	Stomach, esophagus, lung, colorectal,	59	59	59
336		IMH-326	thyroid, kidney normal			
IMH-	Imgenex	N. Adj of	Breast, liver, bladder, ovary, lymph node,	59	59	59
337		IMH-327	pancreas, prostate, uterus, spleen			
IMH-	Imgenex	N. Adj of	Uterus, gall bladder, larynx, uterine cervix,	58	58	58
338		IMH-328	lymph node, colon, vagina, ovary, muscle			
**TMAs with tumor and normal tissues**						
IMH-	Imgenex	Tumor and	Prostate tumor	40	40	49
303		N. Adj	Prostate normal	9	9	
PR-	Biomax	Tumor,	Prostate tumor	50	50	80
807		N. Adj and	Prostate normal	10	10	
		Hyperplasia	Prostate hyperplasia	20	20	
BR-	Biomax	Tumor and	Breast tumors	78	78	80
803		N. Adj	Breast normal	2	2	
CO-	Biomax	Tumor and	Colorectal tumor	81	81	100
1002		N. Adj	Colorectal normal	19	19	
LUC-	Biomax	Tumor,	Lung tumor	36	72	96
961		N. Adj and	Lung normal	4	8	
		Inflammation	Lung inflammatory	8	16	
LVC-	Biomax	Tumor,	Liver tumor	36	72	96
961		N. Adj and	Liver normal	3	6	
		Inflammation	Liver inflammatory	9	18	
KD-	Biomax	Tumor and	Kidney tumor	40	40	48
483		N. Adj	Kidney normal	8	8	
PA-	Biomax	Tumor and	Pancreas tumor	40	40	48
483		N. Adj	Pancreas normal	8	8	
THC-	Biomax	Tumor,	Thyroid tumor	30	60	96
961		N. Adj and	Thyroid normal	3	6	
		Inflammation	Thyroid inflammatory	15	30	

We also used TMA IMH-303 (Imgenex Corp.), containing 40 PCa tissues and 9 matched normal adjacent tissues. Eight additional TMAs from US Biomax were used, each containing multiple tissue specimens of a single tumor type (breast, colon, kidney, lung, liver, pancreas, prostate, thyroid) as well as corresponding disease-free normal control and/or normal adjacent tissues. The manufacturers of these TMAs provided limited basic clinicopathological information (age, sex, tumor stage in some cases) corresponding to the tissue cores, with no patient identifiers. No information was available on patient race or ethnicity, neo-adjuvant treatment, surgical technique, year of surgery, institutions that collected the tissues, number of institutions, follow up routines, and tissue handling techniques. The limited patient follow up data associated with the TMAs prevented any Kaplan-Meier survival analysis.

TMAs were stained using a Biogenic i6000 auto-stainer (Biogenex Corporation, Fremont, CA, USA) as described previously [16], [33]. Briefly, paraffin embedded tissue sections in the TMA slides were deparaffinized and the slides were immersed in Citra-Plus antigen retrieval solution (Biogenex Corp.). Antigen retrieval was performed by microwaving the slides for 2 min at 100% power followed by 10 min at 20% power. Slides were then cooled in the antigen retrieval solution for 20 min. Endogenous peroxidase activity was quenched by treatment with 3% hydrogen peroxide in 10% methanol, and Power Block© universal blocking reagent (Biogenex Corp.) was used to block non-specific protein binding.

TMA slides were incubated overnight with Scripps-Ab5087 anti-LEDGF/p75 antibody [16], followed by 3 washes in PBS. Slides were then incubated with Multi-link© biotinylated secondary antibody (Biogenex Corp.) for 20 minutes, followed by incubation with streptavidin-coupled peroxidase supersensitive Label© (Biogenex Corp.) for 20 min. Immunostaining was detected by peroxidase activation of the 3-amino-9-ethycarbazole (AEC) chromagen (Biocare Medical, Concord, CA, USA). TMAs were counterstained lightly with hematoxylin (Sigma, St. Louis, MO, USA) and mounted with permount (Fisher Scientific, Pittsburgh, PA, USA). For negative control the primary antibody was omitted and substituted with rabbit pre-immune serum. Tissue sections were examined under an Olympus BX50 microscope, and images were acquired using a digital Spot RT3™ camera (Diagnostic Instruments, Sterling Heights, MI, USA). Immunostained TMAs were scored blindly for LEDGF/p75 immunoreactivity by a board certified pathologist. A 4-tier scoring system (0 = negative, 1 = weak, 2 = moderate, 3 = strong) was used to evaluate staining intensity. Tissues with scores of 0–1 were considered to have low intensity staining, whereas tissues with scores of 2–3 were considered to have high intensity staining. Tissue specimens that showed poor quality were excluded from the analyses. These studies were performed under approval by the Institutional Review Board.

### Statistical Analysis

The student's t-test was used to evaluate the significance of the change in mRNA expression between the tumor and normal adjacent control samples in the TissueScan Cancer Q-PCR array. Statistical analysis of IHC data and their relationship to patients' clinical outcomes was done using the SAS software package (version 9.2; SAS institute). For ease of statistical analysis, tissue specimens were grouped into two categories based on their scores. ‘Low’ staining was determined as pooled staining intensity scores of 0 and 1 while ‘high’ staining had pooled scores of 2 and 3. Difference in expression levels of LEDGF/p75 protein between tumors and normal tissues were analyzed using Fisher's exact test.

To control for false positives, which may arise in multiple-hypotheses-testing studies, false discovery rate (FDR) adjusted p-values were also calculated. FDR is a quantification of error used commonly in multiple comparisons. It controls the expected proportion of incorrectly rejected null hypotheses. In other words, FDR controls the fraction of positive detections that are false. Associations between expression levels of LEDGF/p75 and clinicopathological parameters were determined using Fisher's exact test (for age and sex) and Kendall's tau correlation analysis (for tumor stage). Probability values *P*<0.05 were considered statistically significant. Reproducibility of replicate cores was assessed by Cochran-Mantel-Haenszel test which tested for homogeneity between the replicates.

## Results

### LEDGF/p75 transcript is upregulated in various human cancer types

In silico analysis of LEDGF/PSIP1 mRNA expression in cancer tissues was carried out using cancer gene microarray datasets from the Oncomine database that compared cancer tissues to normal tissues (either disease-free normal and/or normal adjacent). As summarized in Table 2, we observed a statistically significant (*P*<0.05) upregulation of LEDGF/PSIP1 transcript in some of the available microarray datasets from cancers of the breast, cervix, colon, esophagus, kidney, head and neck, liver, lung, lymphoma, ovary, pancreas, prostate, salivary gland, skin, and stomach. The fold-increase was greater than 2 in cancers of the breast, cervix, head and neck, kidney, skin, and stomach (Table 2). The increase was modest (between 1 and 2) in colorectal, esophageal, liver, lung, ovarian, pancreatic, prostate, and salivary gland cancers. No changes in LEDGF/PSIP1 transcript expression were detected in bladder (5 datasets) and uterine (1 dataset) cancers. None of the above-mentioned 17 cancer types showed significant downregulation of LEDGF/PSIP1 transcript in any dataset. No datasets were available comparing tumor vs normal expression of LEDGF/PSIP1 transcript in gall bladder, small intestine, and thyroid cancers.

**Table 2:** LEDGF/p75 transcript expression in human tumors determined by analysis of Oncomine cancer gene microarray database.

Cancer type	# of data	% of up-	% of down-	Range fold-change	Range *P* values
	subsets	regulated	regulated		
		subsets	subsets		
Bladder	5	0.0	0	(−1.272) to (1.224)	(0.102) to (0.934)
Breast	17	11.8	0	(−1.449) to (2.557)	(2.29E-18) to (0.956)
Cervix	3	33.3	0	(1.023) to (4.441)	(3.25E-09) to (0.18)
Colorectal	10	30.0	0	(−1.027) to (1.287)	(0.002) to (0.955)
Esophagus	6	16.7	0	(−1.266) to (1.858)	(0.019) to (0.884)
Kidney	14	28.6	0	(−2.521) to (3.512)	(0.001) to (0.944)
Head and neck	12	25.0	0	(−1.124) to (3.692)	(0.001) to (0.995)
Liver	2	50.0	0	(1.043) to (1.38)	(4.15E-5) to (0.265)
Lung	17	11.8	0	(1.89E-06) to (1.00)	(−1.651) to (5.2)
Lymphoma	11	9.1	0	(0.014) to (0.67)	(−1.093) to (2.857)
Ovary	12	8.3	0	(−2.976) to (1.529)	(3.61E-06) to (1.00)
Pancreas	6	16.7	0	(−1.033) to (1.748)	(0.008) to (0.57)
Prostate	13	7.7	0	(−1.88) to (1.272)	(0.017) to (1.00)
Salivary gland	1	100.0	0	1.634	0.001
Skin	10	40.0	0	(−1.392) to (2.014)	(4.67E-06) to (0.859)
Stomach	6	66.7	0	(1.032) to (2.871)	(4.34E-08) to (0.309)
Uterus	1	0.0	0	−1.051	0.617

*P<0.05.

**Figure 1:**
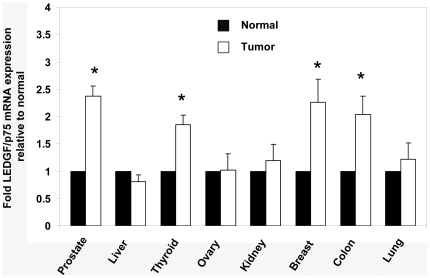
Transcript expression of LEDGF/p75 in eight human cancer types determined by TissueScan Cancer Q-PCR analysis. Data were analyzed using the ΔΔCt method with values normalized to β-actin levels. The y-axis represents the induction fold of the LEDGF/p75 mRNA level in eight cancer types (n = 9) compared to matching normal adjacent tissues (n = 3) in the array. Error bars displays the range of standard error. * *P*<0.05. *P* values were determined with Student's t-test.

The expression of LEDGF/p75 transcript was further assessed experimentally in eight solid tumor types (breast, colon, kidney, liver, lung, ovarian, prostate and thyroid) using the TissueScan Cancer Q-PCR array, which contained cancer tissue samples from 12 individual donor cases (n = 9 for each cancer type, n = 3 for corresponding normal tissues). Primers specific for LEDGF/p75 were used in these experiments. As shown in Figure 1, a statistically significant (*P*<0.05) upregulation of LEDGF/p75 transcript was observed in prostate (2.38 fold, *P* = 0.002), thyroid (1.85 fold, *P* = 0.031), breast (2.26 fold, *P* = 0.037) and colon (2.04 fold, *P* = 0.047) cancers. Although the level of LEDGF/p75 transcript was elevated (>1.02 fold) in other cancer types (with the exception of liver cancer) the fold changes were not statistically significant. Liver tumors showed a slight downregulation (−1.24 fold) in LEDGF/p75 transcript expression, but the decrease was statistically insignificant.

### Selection of LEDGF/p75-Specific Antibody for IHC

**Figure 2:**
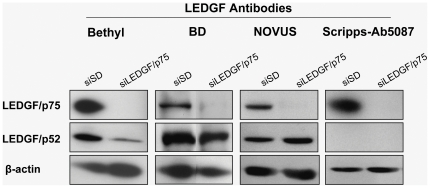
Identification of LEDGF/p75 specific antibody by immunoblotting in PC-3 cells with transient LEDGF/p75 knockdown. Cells were transfected with siLEDGF/p75 to induce transient knockdown of LEDGF/p75. PC-3 cells transfected with small interfering scrambled RNA duplex (siSD) served as corresponding control. Immunoblotting analysis tested the specific reactivity of all the antibodies against LEDGF/PSIP1. All the blot pairs (siSD and siLEDGF/p75) for each antibody were derived from the same blot.

In order to identify an antibody that is specific for LEDGF/p75 and that could be used by IHC to assess the expression levels of this protein in cancer TMAs, we evaluated by immunoblotting the specificity of currently available anti-LEDGF antibodies. Three commercially available anti-LEDGF antibodies (Bethyl, BD and Novus) and a non-commercial antibody (Scripps-Ab5087) against LEDGF were tested by immunoblotting in PC-3 cells with and without transient knockdown of the protein (Figure 2). PC-3 cells transfected with siSD served as control. Immunoblotting analysis indicated that all the 3 commercial antibodies to LEDGF reacted with both p75 and p52 variants. However, the Scripps-Ab5087 antibody detected only LEDGF/p75 and was therefore selected for IHC studies.

### LEDGF/p75 Protein is Overexpressed in Various Human Cancer Types

We performed IHC analysis of LEDGF/p75 protein expression in tumor tissues using a comprehensive panel of TMAs containing a total of 2330 tissue cores, including 1754 tumors tissue cores from over 35 major types of human cancer and corresponding 576 non-tumor (normal and non-cancerous inflammatory) tissue cores (Table 1). We excluded from our statistical analysis those tumor types which had <5 tumor tissue cores or/and had no corresponding non-tumor tissues. The non-tumor inflammatory tissue cores were also excluded because not all tumor types had the corresponding inflammatory tissues. Some tissue cores could not be scored due to poor tissue quality. In the end, 21 tumor types were included in our statistical analysis (Table 3). These comprised a total of 1735 tissues representing single or replicate cores from 1220 individual donor cases, with 985 tumor cases and 235 non-tumor cases.

**Table 3:** Differential expression of LEDGF/p75 protein analyzed by immunohistochemistry in 21 human tumor types and corresponding normal tissues.

Cancer Type	Tumor	Tumor scores (%)		Normal	Normal scores %		*P*
	tissue #	low	high	tissue #	low	high	value
Bladder	55	92.7	7.3	6	83.3	16.7	0.929
Breast	220	79.5	20.5	11	100.0	0.0	0.087
Cervix	10	50.0	50.0	4	100.0	0.0	0.126
Colon	148	75.0	25.0	32	93.8	6.3	**0.012**
Esophagus	10	80.0	20.0	9	22.2	77.8	0.999
Gall bladder	10	90.0	10.0	9	77.8	22.2	0.913
Kidney	122	75.4	24.6	17	70.6	29.4	0.772
Head and neck	17	94.1	5.9	8.0	100.0	0.0	0.680
Liver	100	32.0	68.0	16	68.8	31.3	**0.006**
Lung	164	44.5	55.5	18	55.6	44.4	0.259
Lymphoma	60	85.0	15.0	11	100.0	0.0	0.199
Ovary	76	80.3	19.7	7	100.0	0.0	0.234
Pancreas	105	59.0	41.0	22	54.5	45.5	0.736
Prostate	115	61.7	38.3	30	100.0	0.0	**0.000**
Rectum and anus	7	71.4	28.6	6	50.0	50.0	0.914
Salivary gland	7	71.4	28.6	1	100.0	0.0	0.750
Skin	64	81.3	18.8	6	100.0	0.0	0.309
Small Intestine	5	100.0	0.0	1	100.0	0.0	n/a
Stomach	37	94.6	5.4	13	100.0	0.0	0.544
Thyroid	105	29.5	70.5	16	87.5	12.5	**0.000**
Uterus	43	69.8	30.2	12	100.0	0.0	**0.025**

TMAs stained with antibody against LEDGF/p75 were scored as: 0 = no staining, 1 = low, staining, 2 = moderate staining, 3 = strong staining. Scored tissues were pooled in two groups: low staining (scores 0 and 1) and high staining (scores 2 and 3). *P* values comparing the immunohistochemical expression (high vs. low) of LEDGF/p75 protein between tumor tissues and corresponding normal tissues was determined using the Fisher's exact test. Bold numbers denote significant *P* values. n/a-Not applicable.

**Figure 3:**
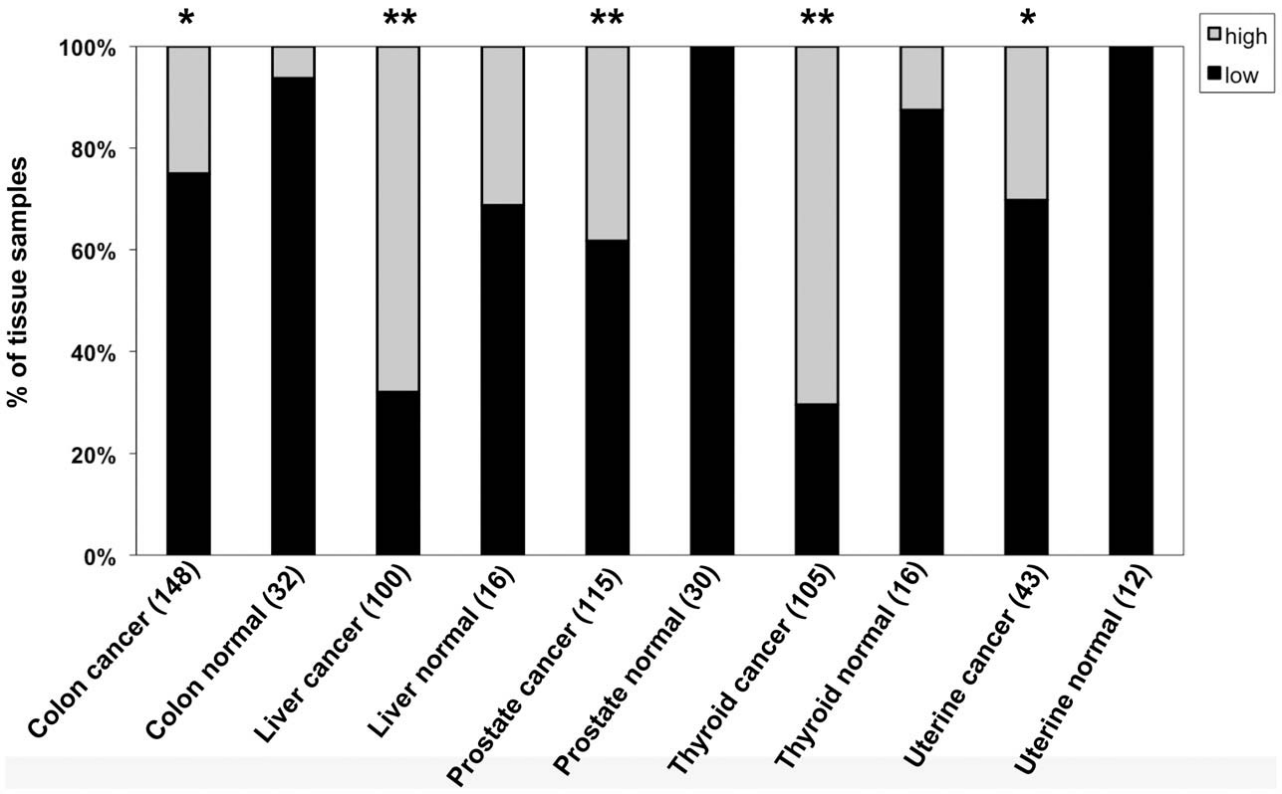
Elevated immunohistochemical expression of LEDGF/p75 protein in five tumor types compared to corresponding normal tissues. Tissue microarrays were stained with antibody against LEDGF/p75, and the individual cores were blindly scored using the following scale: 0 = no staining, 1 = low staining, 2 = moderate staining, 3 = strong staining. Scored tissues were pooled into two groups: low staining (scores 0 and 1, dark bars) and high staining (scores 2 and 3, light bars). The percentage of specimens in the two staining categories was plotted for tumor tissues compared to normal (including disease-free normal and normal adjacent) tissues. *P<0.05; **P<0.01. *P* values were determined with Fisher's exact test.

The results from the IHC analysis of pooled scores for LEDGF/p75 protein expression (i.e., high = scores 2–3 vs low = scores 0–1) in tumor tissues compared to normal tissues are shown in Table 3. There was significant overexpression of the LEDGF/p75 protein in prostate, colon, thyroid, liver, and uterine tumors (*P*<0.05) (Table 3 and Figure 3). Elevated expression levels in breast tumors came close to statistical significance (*P* = 0.087). When corrected for occurrence of false positives, only prostate, thyroid, and liver tumors were found to exhibit significant overexpression of LEDGF/p75 protein (FDR adjusted *P*<0.05).

The percentage of tumor tissues with high (scores 2–3) LEDGF/p75 protein expression was 70.5% in thyroid, 68% in liver, 38.3% in prostate, 30.2% in uterine, and 25% in colon cancers, as compared to 12.5%, 31.3%, 0%, 0%, and 6.3%, respectively, in their corresponding normal tissues (Table 3). There were four cancer types (breast, cervix, ovary, and salivary gland) in which the frequency of tumors displaying high LEDGF/p75 protein expression was about 20% or greater, and the corresponding normal tissues showed little or no expression of the protein. However, when we compared differences in LEDGF/p75 expression (high vs. low expression) between tumor and normal tissues, the *P* values did not reach significance. Interestingly, when we compared differences between tumor and normal tissues with regards to LEDGF/p75 expression (scores 1–3) vs no expression (score 0), the *P* values for breast and cervical cancers reached significance (*P*<0.05; data not shown). Comparison of the replicate cores showed no significant difference (*P* = 0.6226) between the replicates, suggesting high reproducibility between the replicate cores.

**Figure 4:**
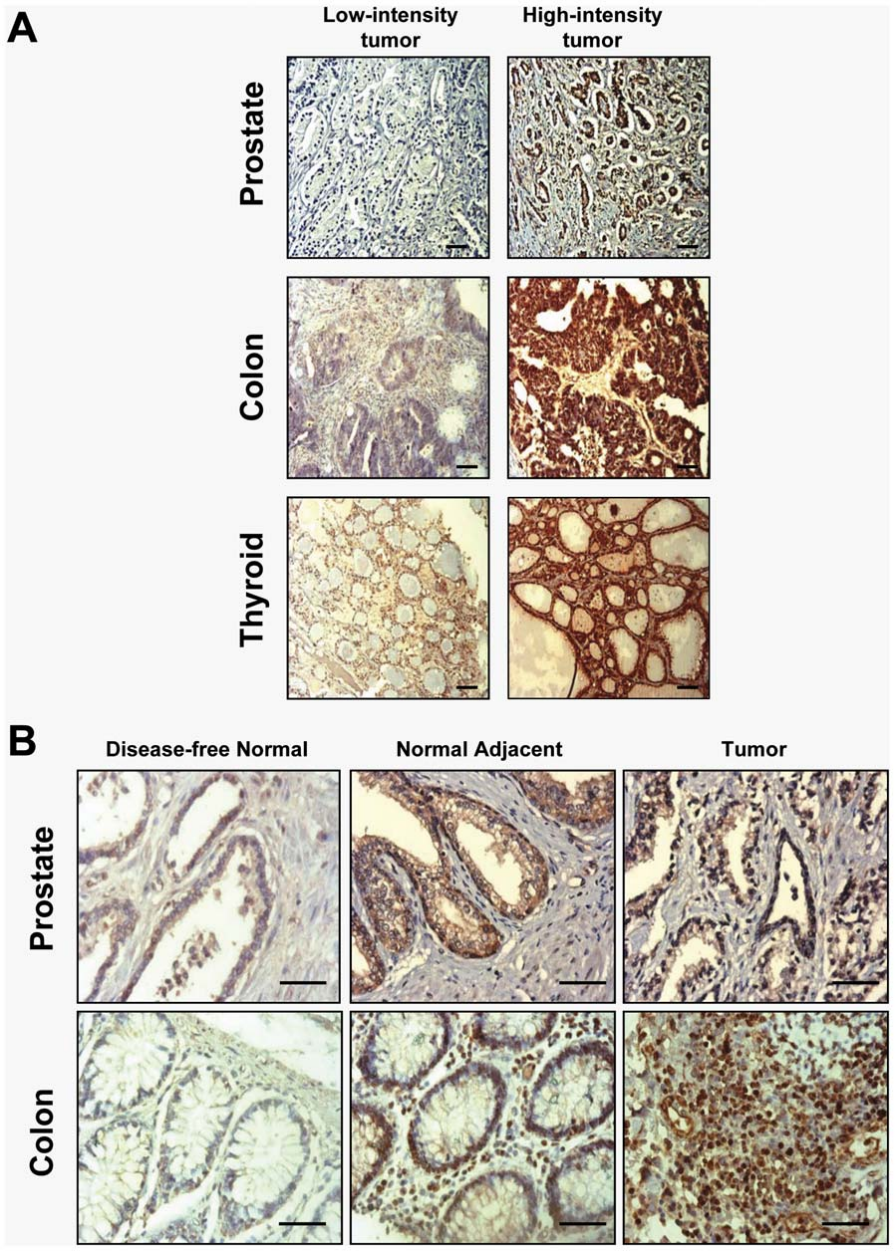
Immunohistochemical staining for LEDGF/p75 protein in selected human tumors. A. Representative images of immunohistochemical staining (low intensity, scores 0–1; high intensity, scores 2–3) for LEDGF/p75 in prostate, colon, and thyroid tumors (Scale bar-40 µm; magnification = 200×). B. Representative images of LEDGF/p75 immunostaining of non-disease normal prostate and colon tissues, and in tumor tissues and their matched adjacent tissues (Scale bar-40 µm; magnification = 400×). TMAs were stained using LEDGF/p75 specific rabbit antibody Scripps-Ab5087, as indicated in Materials and Methods. Identical camera settings were used in the acquisition and processing of images for a particular tissue type.

Figure 4A shows representative tumor tissue cores immunostained with Scripps-Ab5087 anti-LEDGF/p75 antibody. The representative IHC images correspond to prostate, colon, and thyroid tumor tissue cores with low (scores 0–1) and high intensity (scores 2–3) staining. As mentioned above, these three tumor types displayed significant upregulation of both transcript (TissueScan Cancer Q-PCR array) and protein (IHC). Figure 4B shows representative IHC images of disease-free normal prostate and colon tissues (from non-cancer donors), as well as prostate and colon tumor specimens with their matched adjacent tissues. The thyroid cancer TMAs did not have matched adjacent tissues for specific thyroid tumors. We observed that the “normal” tissues adjacent to the prostate and colon tumor tissues had moderate LEDGF/p75 staining, compared to the relatively low intensity staining of the disease-free normal tissues. Another interesting observation was that LEDGFp75 immunostaining was distributed in both the nucleus and the cytoplasm in most tumor tissues examined.

The available clinicopathological characteristics of patients with tumor types displaying significant LEDGF/p75 overexpression are summarized in Table 4. The number of tissue samples for colon and prostate cancers differs in the sets discussing age and sex because of missing data in the commercially available TMAs. Data on age was missing for 4 cores in the prostate cancer TMAs while data on sex was missing for 2 cores in the colon cancer TMA. Correlation between these characteristics and LEDGF/p75 protein expression revealed that overexpression of this protein in liver and thyroid tumors was significantly associated with younger age (*P*<0.05) (Table 4). The median ages of patients represented in the TMAs were 62 years for colon cancer, 53 years for liver cancer, 66 years for prostate cancer, 48 years for thyroid cancer and 70 years for uterine cancer. None of the five tumor types exhibited significant correlation between increased LEDGF/p75 expression, sex or increased TNM tumor stage. The limited patient follow-up data associated with the TMAs precluded correlating LEDGF/p75 expression with patient survival outcomes.

**Table 4:** Association of LEDGF/p75 protein expression in colon, liver, prostate, thyroid and uterine tumors with patients' clinical characteristics.

Characteristic	Colon		Liver		Prostate		Thyroid		Uterus	
	# of	*P*	# of	*P*	# of	*P*	# of	*P*	# of	*P*
	tissues	value	tissues	value	tissues	value	tissues	value	tissues	value
**Age (years)**		0.7045		**0.0473**		0.6646		**0.0283**		0.099
≤Median age	78		50		57		54		18	
>Median age	67		48		54		51		16	
**Sex**		0.5606		0.9771		N/A		0.7579		N/A
Male	97		79		115		41		0	
Female	46		19		0		64		34	
**Tumor stage**		0.4476		0.9182		0.7328		0.1254	N/D	N/A
pT1	2		2		2		4			
pT2	17		54		34		40			
pT3	34		10		45		8			
PT4	9		0		6		4			

Bold numbers denote significant *P* values. *P* values for the association of immunohistochemical LEDGF/p75 expression (high vs. low comparison) in tumors with patients' age and sex were calculated using Fisher's exact test, while those for tumor stage were calculated using Kendall's tau b correlation analysis. At the time of surgical excision of the tissues, the median age of donors was 62 years for colon cancer, 53 years for liver cancer, 66 years for prostate cancer, 48 years for thyroid cancer, and 70 years for uterine cancer. The tumor stages (pT1 to pT4) correspond to pathologic tumor stages T1 through T4 used in the TNM system of cancer staging. The TNM system is based on the extent of the tumor (T), the extent of spread to the lymph nodes (N), and the presence of distant metastasis (M). A number is added to each letter (eg. T1, T2, T3, T4) to indicate the size or extent of the primary tumor and the extent of cancer spread. N/A, Not applicable; N/D, No data available.

The cross-platform analysis of LEDGF/p75 expression in various human tumor types has been summarized in Table 5. The transcript and protein expressions for each tumor type are compared across the different platforms used: in silico, Q-PCR and IHC.

**Table 5:** Summary table of the cross-platform analyses of LEDGF/p75 transcript and protein expression in various human tumor types as compared to corresponding normal tissues.

Cancer type	Oncomine	Q-PCR	Immunohistochemistry
Bladder	X	No data	X
Breast	↑	↑	X
Cervix	↑	No data	X
Colon	↑	↑	↑
Esophagus	↑	No data	X
Gall bladder	No data	No data	X
Kidney	↑	X	X
Head and neck	↑	No data	X
Liver	↑	X	↑
Lung	↑	X	X
Lymphoma	↑	No data	X
Ovary	↑	X	X
Pancreas	↑	No data	X
Prostate	↑	↑	↑
Rectum and anus	↑	No data	X
Salivary gland	↑	No data	X
Skin	↑	No data	X
Small Intestine	No data	No data	X
Stomach	↑	No data	X
Thyroid	No data	↑	↑
Uterus	X	No data	↑

↑, Statistically significant (p<0.05) increase in LEDGF/p75 expression in tumors versus normal tissues;

X, No statistically significant (p<0.05) change in LEDGF/p75 expression in tumors versus normal tissues; No data, no datasets or tissues available.

## Discussion

The present study was undertaken as part of our ongoing efforts to understand the biological and clinical significance of LEDGF/p75 expression in human cancer. Our results indicated significant upregulation of both LEDGF/p75 transcript and protein in prostate, colon, and thyroid tumors, inferred by the analysis of transcript expression in the Oncomine cancer gene microarray database (when data available) and the TissueScan Cancer Q-PCR array, and analysis of protein expression by IHC in TMAs. The observed upregulation of LEDGF/p75 in prostate tumors is consistent with our previous reports that this protein is the target of autoantibodies in some patients with PCa, is upregulated in both PCa cell lines and tissues, and promotes chemoresistance in PCa cell lines when ectopically overexpressed [16], [31].

Significant upregulation of LEDGF/PSIP1 transcript was also observed in some Oncomine datasets for cancers of the breast, cervix, esophagus, kidney, head and neck, liver, lung, lymphoma, ovary, pancreas, salivary gland, skin, and stomach. However, only 4 out of 8 tumor types (prostate, colon, breast and thyroid) exhibited significant upregulation of the LEDGF/p75 transcript in the TissueScan Cancer Q-PCR array. Although other tumor types (kidney, lung, and ovarian) exhibited >1.02 fold LEDGF/p75 transcript elevation in this array, the upregulation was not statistically significant. These differences between the Oncomine database and the TissueScan Cancer Q-PCR array could be attributed to the small sample size in the latter, methodological/platform variations, analysis of LEDGF/PSIP1 vs LEDGF/p75, and the use of unrelated tumor data sets.

In addition to prostate, colon and thyroid cancers, the LEDGF/p75 protein levels were significantly elevated, as assessed by IHC analysis of TMAs, in liver and uterine cancers. To control for false positives, which are expected when numerous independent statistical tests are performed, FDR analysis was done. The FDR adjusted p-values suggested that the LEDGF/p75 protein overexpression in prostate, thyroid, and liver tumors was not by chance. Elevated expression of LEDGF/p75 in liver and thyroid tumors was associated with younger age, while none of the five tumor types displayed a significant correlation between increased LEDGF/p75 expression and increased tumor stage, most likely due to the relatively small number of tumor samples in stages pT1 and pT4.

An interesting observation in our IHC analysis was that LEDGF/p75 was not exclusively confined to the nucleus, but often appeared in the cytoplasm of tumor cells. We cannot rule out the possibility that this could be due to non-specific reactivity of the Scripps-Ab5087 anti-LEDGF/p75 antibody. However, this would seem unlikely given that the antibody reacted specifically with LEDGF/p75 in immunoblots, and the cytoplasmic staining was not observed in all tissues, particularly in normal tissues. It could be speculated that depending on the particular tumor microenvironment, LEDGF/p75 could shuttle between the extracellular milieu, the cytoplasm, and the nucleus, as observed for other stress or survival proteins [34]–[36]. While there is compelling evidence that LEDGF/p75 exhibits a nuclear localization in cultured cell lines, early studies suggested that the protein exists as both endogenous and secreted forms [4], [37]. It would be important to determine in future studies whether LEDGF/p75 is secreted from tumor cells, and if this release provides a survival advantage to cancer cells in a stressful tumor microenvironment.

We did not observe a statistically significant overexpression of LEDGF/p75 protein in bladder tumors, which is inconsistent with a previous report by Daugaard et al. [17] showing upregulation of LEDGF/p75 mRNA in bladder tumors from Affymetrix microarray data. None of the 5 Oncomine bladder cancer datasets analyzed for LEDGF/PSIP1 transcript expression showed significant upregulation. However, our data on elevated transcript expression of LEDGF/p75 in breast tumors was in agreement with that of Daugaard et al. [17]. Our analysis showed significant upregulation of LEDGF/PSIP1 transcript levels in breast tumors in 2 of the 17 Oncomine breast cancer gene microarray datasets, and robust elevation of LEDGF/p75 transcript (2.26 fold, *P* = 0.037) in the TissueScan Cancer Q-PCR array. Likewise, our evaluation of LEDGF/p75 protein levels in the breast tumor tissues by IHC came very close to being statistical significant (*P* = 0.087). Daugaard et al. [17] also reported lack of LEDGF/p75 transcript upregulation in colon cancer, whereas we found significant transcript upregulation in 3 of 10 Oncomine colorectal cancer datasets, and in the TissueScan Cancer Q-PCR array. Our IHC analysis also indicated LEDGF/p75 overexpression in colon cancer. These discrepancies between our results and those of Daugaard et al. [17] might be related to differences in the patient cohorts or in the methodologies/platforms used.

A limitation of the present study is that the analyses of LEDGF/p75 transcript and protein expression were done in unrelated tumor datasets. It should be emphasized, however, that this study was designed to determine if LEDGF/p75 is a commonly upregulated protein in major human cancers. Our results indicate that LEDGF/p75 upregulation in human cancer is selective, with statistically significant elevation of both transcript and protein in prostate, colon, and thyroid tumors, as determined by TissueScan Cancer Q-PCR arrays and IHC analysis of TMAs. However, we observed a lack of transcript-protein correlation for the other cancer types. While this is likely due to the use of unrelated tumor samples in the transcript and protein analyses, we cannot rule out that in certain tumors the expression of LEDGF/p75 transcript does not correlate with protein expression. There are several reports documenting this phenomenon for other cancer-associated proteins [33], [38]–[44]. For instance, integrative studies of transcript and protein expression profiles in tumors and cancer cell lines showed discordant protein and mRNA expression, revealing a 20%–65% concordance depending on the tumor type [41]–[43]. As a specific example, transcript expression of the Pdc4 tumor suppressor gene was found suppressed in tumors compared to normal tissues, but the protein levels were elevated [39]. Likewise, it was observed that in nasopharyngeal carcinomas, the LMP1 mRNA and protein expression levels did not correlate [44]. Factors leading to discordant transcript-protein correlation in tumor cells include abnormal protein stabilization via protein-protein interactions or post-translational modifications, differential lifetimes of the mRNA and protein species, mRNA regulation by miRNAs, differential regulation or processing of protein isoforms, and other mechanisms not yet completely understood [38]–[44].

Our results highlight the need for analyzing simultaneously LEDGF/p75 mRNA and protein expression in the same tumor samples in future studies aimed at investigating the role of this pro-survival protein in the progression of a specific cancer type, and its potential value as a biomarker for tumor aggressiveness or chemoresistance. Such studies should involve a large patient sample size with complete annotated clinical data, as well as inclusion of several disease-free normal control tissues. Indeed, a limiting factor in our studies, and in most studies comparing gene expression in tumor vs normal tissues, is that the control tissues analyzed are often histologically/morphologically “normal” tissues adjacent to the tumors. These “normal adjacent” tissues are susceptible to “field cancerization”, an effect that has been documented in many human tumor types and that involves molecular abnormalities in the normal adjacent tissue such as increased expression of cancer-associated genes or proteins, changes in gene methylation patterns, microsatellite alteration, increased oxidative DNA damage and angiogenesis, and TMPRSS2-ERG-fusions [45]–[50]. Field cancerization associated with increased oxidative stress and inflammation in areas surrounding the tumors could lead to upregulation of stress response proteins such as LEDGF/p75 in the normal tissue adjacent to the tumor, resulting in underestimation of the extent of its overexpression in the tumors. Consistent with this notion, we reported recently that the expression levels of the stress/antioxidant proteins peroxiredoxins 3 and 4 in normal tissues adjacent to prostate tumors are intermediate between tumor tissues and non-diseased normal prostate tissues [33].

To our knowledge, this is the first comprehensive survey of the expression of LEDGF/p75 transcript and protein in human cancers. In summary, our results revealed a significant upregulation of LEDGF/p75 (transcript or protein) in several cancer types, particularly in prostate, breast, colon, liver, thyroid and uterine malignancies (summarized in Table 5). Because our study was limited by the lack of complete clinical and follow-up patient data associated with the TMAs, the prognostic significance of LEDGF/p75 overexpression in human cancer still remains unclear. Further studies analyzing simultaneously both LEDGF/p75 transcript and protein in the same tissues, using large patient cohorts with complete clinical and follow-up data, are necessary to determine if LEDGF/p75 upregulation correlates with tumor progression and aggressiveness in a specific cancer type. Taken together, the results presented here further establish LEDGF/p75 as a cancer-related protein, and strengthen the rationale for investigating the biological and clinical significance of its upregulation in human tumors.
